# Improving the Microstructure and Mechanical Properties of Al-0.7Fe-0.4Mg-0.1Si-0.5Er Alloy by Equal Channel Angular Pressing

**DOI:** 10.3390/ma18174007

**Published:** 2025-08-27

**Authors:** Xingchi He, Fuyu Dong, Yue Zhang

**Affiliations:** School of Materials Science and Engineering, Shenyang University of Technology, Shenyang 110870, China; xingchi_he@smail.sut.edu.cn (X.H.);

**Keywords:** micro-alloying aluminum alloy, equal channel angular pressing, homogenization annealing, tensile strength, electrical conductivity

## Abstract

The ECAP (equal channel angular pressing) technique plays a crucial role in enhancing the overall performance of aluminum alloys. In this study, ECAP was applied to a self-developed micro-alloyed Al-0.7Fe-0.4Mg-0.1Si-0.5Er aluminum alloy to investigate the strengthening effects of varying numbers of passes. The results show that after four ECAP passes, the alloy achieved a high tensile strength (208 MPa), yield strength (175.4 MPa), elongation after fracture (10.8%), and a relatively high electrical conductivity (57.1%IACS). The enhanced strength is primarily attributed to precipitation strengthening ($\sigma_{p}$), grain refinement strengthening ($\sigma_{gbs}$), and dislocation strengthening ($\sigma_{dis}$). The grain refinement is a result of dynamic recrystallization (DRX) induced by severe plastic deformation. This study demonstrates that ECAP enables a significant improvement in the mechanical properties (82.3%) of the alloy while causing only a marginal reduction (2.9%) in electrical conductivity. These findings provide both technological and theoretical support for the manufacturing of high-performance conductors and other lightweight electrical structural components.

## 1. Introduction

With the extension of power transmission distances and the continuous growth in electricity demand, existing conductor materials face numerous challenges, including insufficient mechanical strength and reduced electrical conductivity under service conditions [1,2]. Due to their low density, good electrical conductivity, and relatively low cost, aluminum alloys have emerged as a promising alternative to conventional copper conductors [3]. However, to meet the increasing demands for higher strength and operational stability, conventional aluminum alloy systems are no longer sufficient for the development of high-performance conductors and other lightweight electrical structural components.

In recent years, micro-alloying technology has been widely applied in the study of performance enhancement for aluminum alloys [4,5]. The introduction of trace elements into the aluminum matrix can effectively refine grains and suppress recrystallization, thereby enhancing strength without significantly compromising electrical conductivity [6]. However, it is worth noting that the extremely low addition levels of micro-alloying elements impose inherent limitations on the extent of performance enhancement [7]. Meanwhile, micro-alloyed aluminum systems still exhibit insufficient microstructural stability, indicating the need for further improvement [8]. Although heat treatment can regulate the types and distribution of precipitates in the alloy and thereby further strengthen the material, it often induces grain coarsening [5,9]. Therefore, heat treatment alone is insufficient to satisfy the concurrent requirements for strength and electrical conductivity in power conductors.

Equal channel angular pressing (ECAP) is a severe plastic deformation technique capable of producing significant grain refinement and increasing dislocation density, thereby markedly enhancing material strength with minimal impact on electrical conductivity. This technique shows great potential optimizing the comprehensive properties of aluminum alloy. For example, Cardoso et al. [10] processed AA7075 aluminum alloy via ECAP and achieved substantial improvements in both tensile properties and microhardness. Surendar Nath et al. [11] enhanced the mechanical and corrosion resistance of commercially pure aluminum using a self-designed ECAP tool. Maged Elhefnawey et al. [12] demonstrated that the refinement of microstructure and enhancement of strength induced a transformation in the wear mechanism after ECAP passes of AA7075 alloys. Meanwhile, ECAP processing parameters are known to be strongly correlated with the mechanical properties of the material, mainly due to variations in the microstructural evolution mechanisms after each pass. Liu and Jia [13,14] further demonstrated that varying the number of ECAP passes can refine grains, homogenize the microstructure, and promote the segregation of precipitate phases. Therefore, optimizing ECAP parameters is crucial for tailoring microstructures and improving alloy performance.

In our previous study [15], a novel micro-alloyed Al-0.7Fe-0.4Mg-0.1Si-0.5Er alloy was developed by adding Er, achieving a tensile strength of 135.3 MPa and an electrical conductivity of 53.3%IACS. However, this performance was still insufficient for long-distance, high-capacity power transmission conductors. While ECAP has been extensively studied, most works have focused on single-pass processing, other rare-earth additions, or mechanical properties alone, leaving the combined effects of Er micro-alloying and multi-pass ECAP on both mechanical and electrical properties largely unexplored. Therefore, an ECAP tool was designed to further improve the mechanical properties of the Al-0.7Fe-0.4Mg-0.1Si-0.5Er alloy. In this study, we systematically investigate the effects of ECAP pass number on grain size, recrystallization mechanisms, precipitate characteristics, and precipitation locations, along with tensile properties, microhardness, and electrical conductivity. Based on these results, we elucidate the strengthening mechanisms involved and identify the optimal number of passes for balancing strength and electrical conductivity in this alloy system.

## 2. Materials and Methods

### 2.1. Materials and ECAP Processing

The base material used in this study was an Al-0.7Fe-0.4Mg-0.1Si-0.5Er alloy. It was prepared by melting at 650 °C using high-purity aluminum (99.99%), pure magnesium (99.9%), high-purity zinc (99.99%), and master alloys of Al-20%Fe, Al-50%Cu, Al-20%Si, Al-3%B, and Al-20%Er (all supplied by Inner Mongolia JinLian Aluminum Co., Ltd., Inner Mongolia, China). Unless otherwise specified, the alloy compositions in this study are given in wt.%. The actual chemical composition of the alloy was determined by inductively coupled plasma–optical emission spectroscopy (ICP-OES, Agilent 5110, Agilent Technologies, Santa Clara, CA, USA), and the results are shown in Table 1.

**Figure 1:**
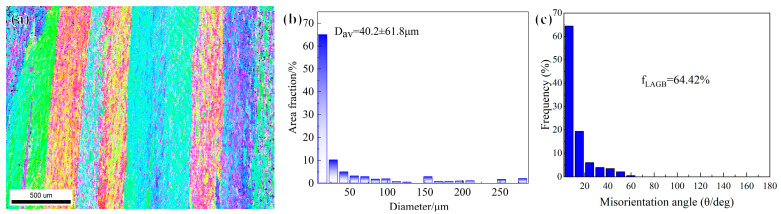
Electron backscatter diffraction (EBSD) results of homogenization annealing alloy: (**a**) grain morphology, (**b**) average grain size, and (**c**) misorientation angle.

Subsequently, a homogenization annealing treatment was conducted at 550 °C for 1 h in an air atmosphere at ambient pressure, achieving a tensile strength of 114.1 MPa and an electrical conductivity of 58.8%IACS [15]. As shown in Figure 1, the homogenization-annealed alloy exhibits large grains with an average size of 40.2 μm and a high-angle grain boundary (HAB) fraction of 64.42%. The parallel color bands in the IPF map correspond to crystallographic orientation bands inherited from the as-cast structure, and the grains are defined by the HABs marked in black.

**Figure 2:**
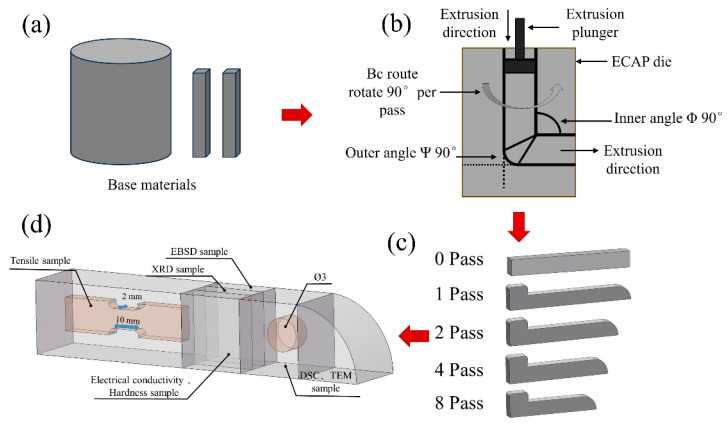
ECAP processing procedure and sample extraction locations for testing: (**a**–**c**) ECAP processing procedure and (**d**) sample extraction locations.

The ECAP processing procedure and sample extraction locations for testing are shown in Figure 2. Prior to ECAP processing, the base material was machined into rectangular billets with dimensions of 10 mm × 10 mm × 120 mm (Figure 2a). All six faces of each billet were polished with 160 # abrasive paper to ensure surface quality and reduce the risk of crack initiation during deformation. ECAP experiments were performed using a die with an inner channel angle (Φ) and the outer arc angle (ψ) both set to 90°. Before pressing, a lubricant composed of molybdenum disulfide and engine oil was applied to the die channels, and the billets were wrapped in graphite paper to minimize friction during extrusion. The processing experiments were conducted on an MTS Exceed E45 universal testing machine with a maximum load of 500 kN at a constant ram speed of 5 mm/min. ECAP samples were prepared with 1, 2, 4, and 8 passes, respectively. The locations for mechanical/electrical testing and microstructural characterization are illustrated in Figure 2d.

### 2.2. Testing Instruments

The electron backscatter diffraction (EBSD) analysis was performed using a Gemini SEM 300 field emission scanning electron microscope (Carl Zeiss AG, Oberkochen, Germany) to statistically analyze grain orientation, grain size, and grain morphology. Prior to EBSD, samples were mechanically ground using SiC papers ranging from 600 # to 2000 #, followed by electrolytic polishing. Electropolishing was performed at 18 V for 20 s in an electrolyte consisting of 3.5% perchloric acid, 84% methanol, and 12.5% glycerol at 23 °C. Transmission electron microscopy (TEM) was conducted using an FEI Talos F200X microscope to characterize the morphology, composition, location, size, and crystal structure of the precipitates. TEM foils were prepared by wire-cutting 3 mm diameter and 500 μm thick discs, which were then ground to ~70 μm thickness using 1000 #, 1500 # and 2000 # SiC papers. Final thinning was achieved by twin-jet electropolishing in a 1:3 nitric acid/methanol solution at 14 V and −20 °C. Ion milling was subsequently performed at 5 keV and ±5° for 15 min, followed by low-voltage polishing at 4 keV and ±4° until an electron-transparent area was obtained. X-ray diffraction (XRD) analysis was carried out using a SHIMADZU XRD-7000 diffractometer (Shimadzu Corporation, Kyoto, Japan) with Cu-Kα radiation to identify phases and analyze crystallographic texture. Scans were performed over a 2θ range of 20–100° at a scanning rate of 8°/min. Fracture surface morphology after room-temperature uniaxial tensile testing was examined using a scanning electron microscope (SEM, Hitachi High-Technologies Corporation, Tokyo, Japan).

Mechanical and electrical properties were evaluated as follows. Vickers hardness (HV) was measured using an HVS-5 hardness tester (Shanghai Taiming Optical Instrument Co., Ltd., Shanghai, China) under a load of 100 g with a dwell time of 10 s. Tensile tests were performed on a CMT5105 electronic universal testing machine (MTS Systems Co., Ltd., Shenzhen, China) at an initial strain rate of 10^−3^ s^−1^. Specimens were prepared in accordance with ASTM E8, and three samples were tested for each condition. The electrical conductivity of the ECAP-processed samples was measured using a DK60K digital eddy current conductivity meter (Beijing United Test Co., Ltd., Beijing, China). Each sample was tested five times, and the average value was reported.

## 3. Results and Discussion

### 3.1. Effect of ECAP on Phase Evolution

**Figure 3:**
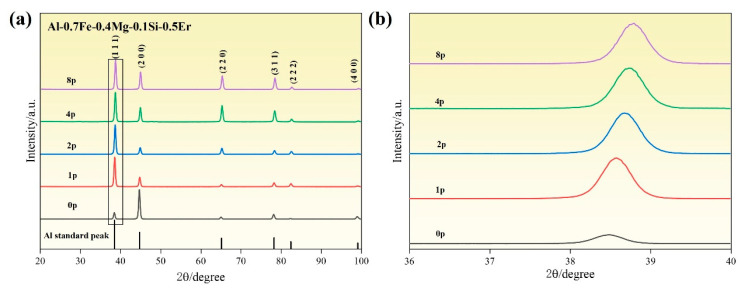
XRD results of Al-0.7Fe-0.4Mg-0.1Si-0.5Er alloy after different ECAP passes: (**a**) XRD results and (**b**) enlarged view of the (111)_Al_.

Figure 3 presents the XRD patterns of the alloys subjected to different passes of ECAP. In all conditions, only the characteristic peaks of the α-Al matrix are detected, with no apparent emergence of new diffraction peaks. This is attributed to the very limited amount of precipitate particles formed by micro-alloying, which makes them difficult to detect. Although no additional peaks appear, a redistribution of relative peak intensities is observed after ECAP. This is mainly due to textural evolution during severe plastic deformation, which changes the proportion of grains in specific crystallographic orientations, thereby modifying the relative intensities of corresponding diffraction planes. As shown in the enlarged view of the (111)_Al_ plane (see Figure 3b), the diffraction peak gradually shifts to the right, and the full width at half maximum (FWHM) increases with the passes of ECAP. This behavior is attributed to the accumulation of high strain—which distorts the aluminum lattice and induces lattice expansion [16]—and to the high dislocation density generated during severe plastic deformation, which increases microstrain within the grains. The latter is consistent with TEM observations showing dense dislocation networks and subgrain boundaries. In addition, the broadening of the diffraction peaks further supports the occurrence of lattice distortion during the ECAP process [17].

### 3.2. Effect of ECAP on Grain Morphology

**Figure 4:**
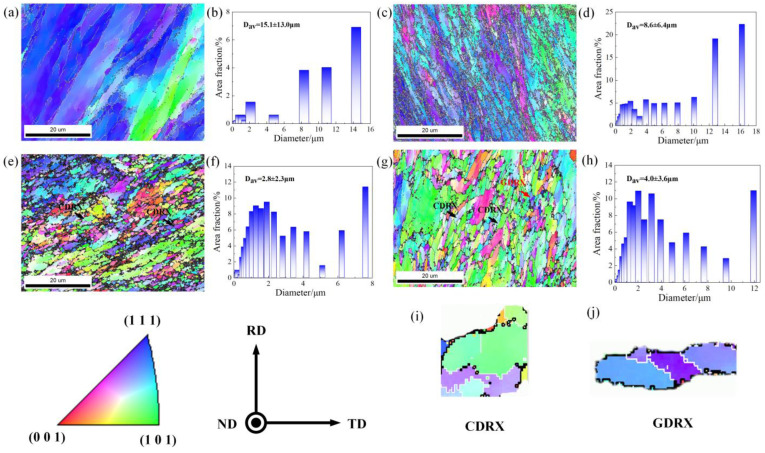
Grain morphology and average grain size after different ECAP passes: (**a**,**b**) 1p, (**c**,**d**) 2p, (**e**,**f**) 4p, (**g**,**h**) 8p, (**i**) CDRX, (**j**) GDRX.

Figure 4 illustrates the grain morphologies obtained after different ECAP passes, where black lines indicate high-angle grain boundaries (HABs) and white lines represent low-angle grain boundaries (LABs). Overall, ECAP processing leads to a noticeable evolution in grain orientation and a reduction in average grain size. It can be seen in the 1p that the grains are elongated with a pronounced (111) texture and an average size of approximately 15.1 ± 13.0 μm. After two passes, the grains remain elongated but are markedly refined, with an average size of about 8.6 ± 6.4 μm. In addition, the dominant orientations are (111) and (101), and the texture intensity is weakened. Notably, fine dispersoids are observed along the grain boundaries, which are presumed to be precipitate particles such as Mg_2_Si or Al_13_Fe_4_. The elongation of grains in the early passes is mainly caused by intense shear along the ECAP shear plane.

After four passes, the elongated grains are transformed into elliptical block-like grains, and the average grain size is further reduced to 2.8 ± 2.3 μm. Meanwhile, a necklace-like structure composed of alternating LABs and HABs (see Figure 4i) indicates the occurrence of dynamic recrystallization (DRX) during processing [18,19]. Additionally, the dispersed precipitate particles tend to agglomerate. At this stage, continuous dynamic recrystallization (CDRX) progressively converts LABs into HABs, promoting the transition from elongated grains to near-equiaxed morphologies. When the ECAP passes are further increased to eight, the microstructure becomes dominated by equiaxed elliptical grains with an average size of 4.0 ± 3.6 μm, indicating grain coarsening. The complete transformation to equiaxed grains is attributed to the combined effects of CDRX and geometric dynamic recrystallization (GDRX), the latter being promoted by grain subdivision and rotation under high accumulated strain. The occurrence and extent of DRX are also dependent on deformation temperature and strain rate; higher temperatures and lower strain rates facilitate grain boundary migration and grain growth, whereas lower temperatures and higher strain rates, as used in this study, favor finer DRX grains due to suppressed recovery and limited growth time. Evidence of GDRX is highlighted in Figure 4j and is mainly attributed to the higher stored strain energy after eight passes [20,21]. Meanwhile, the amount of precipitate particles near the grain boundaries decreases, because the greater mechanical work in the eight-pass route converts more mechanical energy into internal energy, driving secondary dissolution of the particles during DRX [22].

**Figure 5:**
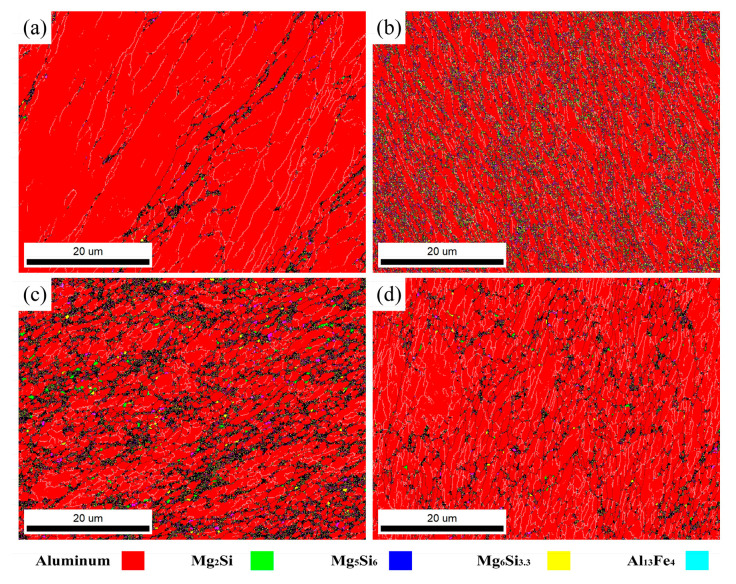
Phase distribution under different ECAP passes: (**a**) 1p, (**b**) 2p, (**c**) 4p, (**d**) 8p.

The above results indicate that the passes of ECAP significantly affect the distribution of precipitates. Specifically, the precipitate content increases during the early ECAP passes and decreases at higher passes. EBSD-based quantitative analysis (Figure 5) reveals this trend over large scanned areas; however, several dark regions appear in the maps, which are challenging to index due to the small scale of precipitates and significant plastic strain. This limitation is related to the spatial resolution of EBSD, which may underestimate submicron-scale particles. TEM observations () confirm the presence of precipitates typically a few hundred nanometers in size, located preferentially along HABs and, to a lesser extent, within grains. Their morphologies are consistent with the Mg_2_Si and Al_13_Fe_4_ phases identified in EBSD phase maps [15], although precise phase identification from TEM alone is not conclusive in this study.

In the early ECAP passes, severe plastic deformation introduces a high density of dislocation tangles, which tend to accumulate near grain boundaries and provide abundant nucleation sites for precipitation, resulting in a higher precipitate density. These newly formed precipitates are submicron-scale and uniformly distributed. At eight passes, the slightly lower precipitate content compared to the four-pass sample is attributed to the combined effects of precipitate fragmentation, partial dissolution during dynamic recrystallization, and coarsening driven by deformation heating and strain energy release [23]. Some precipitates also exhibit irregular morphologies due to deformation-induced fragmentation, but the dominant types (Mg_2_Si and Al_13_Fe_4_) remain unchanged throughout the process. The precipitates are predominantly fine particles, mainly located near HABs, with only a small portion present within the grains. Table 2 presents the fractions of precipitates after different ECAP passes showing that the Mg_2_Si and Al_13_Fe_4_ phases exhibit similar evolution behavior, with the highest fraction of dispersed precipitates (approximately 15.5%) observed after four ECAP passes. Therefore, EBSD and TEM provide complementary perspectives; EBSD offers statistical coverage over large areas, while TEM confirms the presence, size, and distribution of submicron precipitates that may be underestimated in EBSD maps, together supporting the observed precipitation trend.

**Figure 6:**
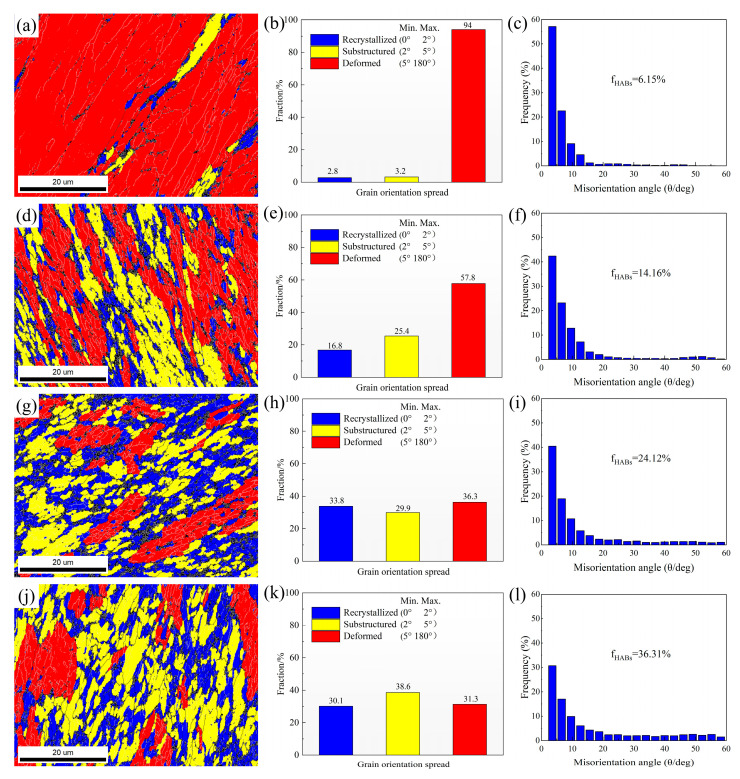
GOS maps and misorientation angle of different ECAP passes: (**a**–**c**) 1p, (**d**–**f**) 2p, (**g**–**i**) 4p, (**j**–**l**) 8p.

To further investigate the effect of ECAP passes on recrystallization behavior, GOS maps were used for quantitative analysis, as shown in Figure 6. After one pass, most grains remained in a deformed state, with recrystallized and substructured grains accounting for only 6%. With an increasing number of ECAP passes, the fraction of recrystallized and substructured grains gradually increased. As shown previously in Figure 4, this trend is attributed to the DRX induced by repeated ECAP deformation. The distribution of misorientation angles reveals that the fraction of HAGBs increases with the number of ECAP passes. This is primarily due to the progressive transformation of low-angle grain boundaries into high-angle ones during the DRX process [24].

According to previous studies, DRX facilitates strain energy release and promotes microstructural homogeneity. However, each ECAP pass introduces additional strain into the material. Even after DRX, a significant amount of stored strain energy remains within the alloy. This stored energy, originating from dislocation density and lattice distortion, provides the driving force for grain boundary migration, the transformation of LABs into HABs, and the refinement of grain structure during multi-pass ECAP. The relationship between strain energy (*ε*) and geometric required dislocation (GND) density can be expressed as follows [25]:

(1) $$\varepsilon =\frac{1}{2}G\rho b^{2}$$

where the *G* is the shear modulus, *b* is the Burgers, and ρ is the GND density. Meanwhile, the GND density can be expressed as

(2) $$\rho \approx \frac{\alpha \theta }{bu}$$

(3) $$\theta =\frac{\beta }{180}\pi$$

where α is a constant value, θ (rad) is the average misorientation angle across the dislocation boundaries, u is the step size of the EBSD map, and β is the KAM value.

Based on this, the relationship between stored energy (*ε*) and KAM can be expressed as follows:

(4) $$\varepsilon =\frac{1}{360}Gb\frac{\alpha \theta \pi }{u}\beta$$

**Figure 7:**
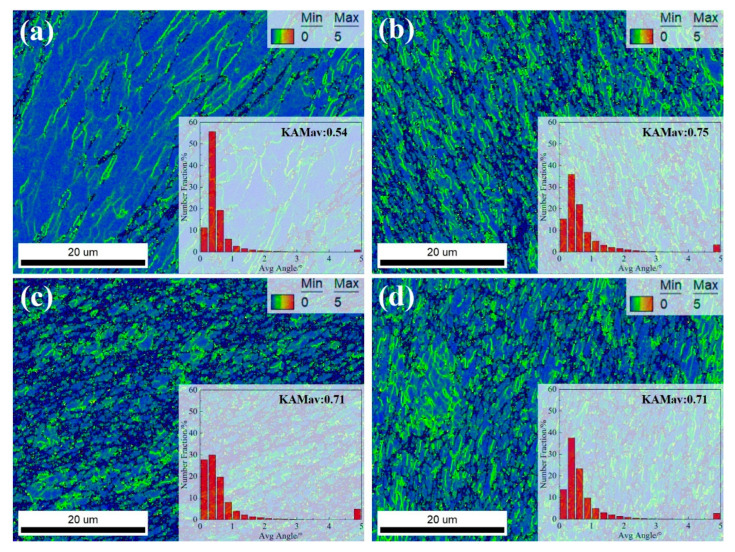
Maps of the kernel average misorientation under different ECAP passes: (**a**) 1p, (**b**) 2p, (**c**) 3p, (**d**) 4p.

Therefore, kernel average misorientation (KAM) analysis was applied to these samples processed with different ECAP passes, as shown in Figure 7. It can be observed that after 1 pass, although the internal grains mainly exhibit deformed structures, the kernel average misorientation (KAM) value remains low, at only 0.54. After two passes, dynamic recrystallization occurs within the alloy; however, the KAM value increases to 0.75, indicating a rise in local strain gradients [26]. With further increases in the number of ECAP passes, the KAM values decrease to 0.71 and 0.71 for four and eight passes, respectively. It can be concluded that the internal stored energy initially increases and then decreases with the increasing number of ECAP passes. It is worth noting that the KAM reflects the dislocation structure within the alloy after ECAP deformation [27]. Specifically, after a single ECAP pass, although the alloy undergoes severe plastic deformation, the internal dislocation density remains relatively low. As the number of ECAP passes increases, although DRX promotes dislocation annihilation, the accumulation of dislocations caused by plastic deformation outweighs their elimination. When the number of passes exceeds four, the KAM values remain nearly constant, indicating a dynamic equilibrium between dislocation generation and annihilation.

**Figure 8:**
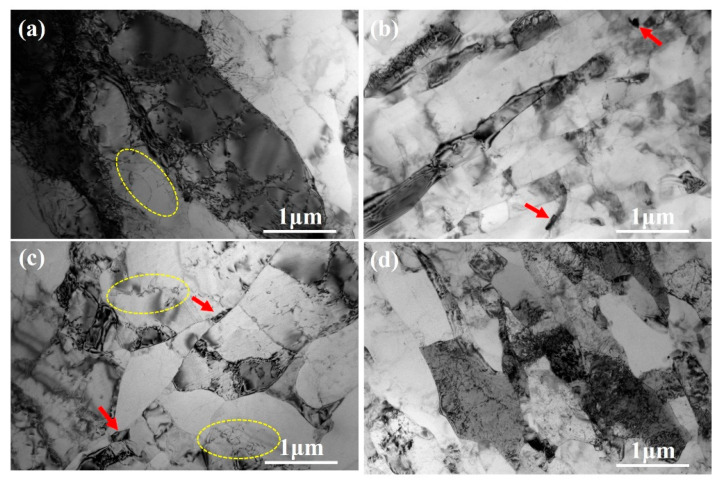
TEM bright-field images of the ECAP samples: (**a**) 1p, (**b**) 2p, (**c**) 4p, (**d**) 8p.

Figure 8 presents the TEM bright-field images of the alloys subjected to different passes of ECAP. In Figure 8a (1 pass), the grains exhibit elongated morphologies and relatively large sizes, with dislocations visible within the grains (yellow ellipses). In addition to isolated dislocations, localized dislocation clusters and wall-like arrangements can also be discerned, reflecting the early stages of dislocation rearrangement. Based on direct measurements from representative images, a few precipitate particles are observed, typically with sizes of 200–500 nm, and occasionally up to about 800 nm. After two passes (Figure 8b), the grains are significantly refined but still retain an elongated shape, and a few equiaxed grains also appear. A small number of precipitate particles are present within the grains (red arrows), with sizes generally within the same range as in Figure 8a. More pronounced dislocation networks, including clusters and wall-like configurations, are observed, indicating enhanced dislocation interactions and the development of subgrain boundaries. When the number of passes exceeds four (Figure 8c,d), the grains become further refined and predominantly equiaxed. Fine precipitate particles are concentrated near the grain boundaries, while dislocation structures are still present within the grains. The synergistic effect of these phenomena, even at small volume fractions, significantly contributes to the enhancement of strength, as demonstrated by Sauvage et al. [28]. In addition, some fine grains are observed near the grain boundaries. Although a complete statistical size distribution from TEM was not feasible due to the limited number of suitable images and the difficulty of unambiguously identifying certain phases, the observed precipitate size range is consistent with the scale inferred from EBSD analysis. These TEM observations provide conclusive evidence supporting the EBSD analysis of grain morphology and precipitate distribution.

### 3.3. Effect of ECAP on Mechanical Properties

#### 3.3.1. Microhardness

**Figure 9:**
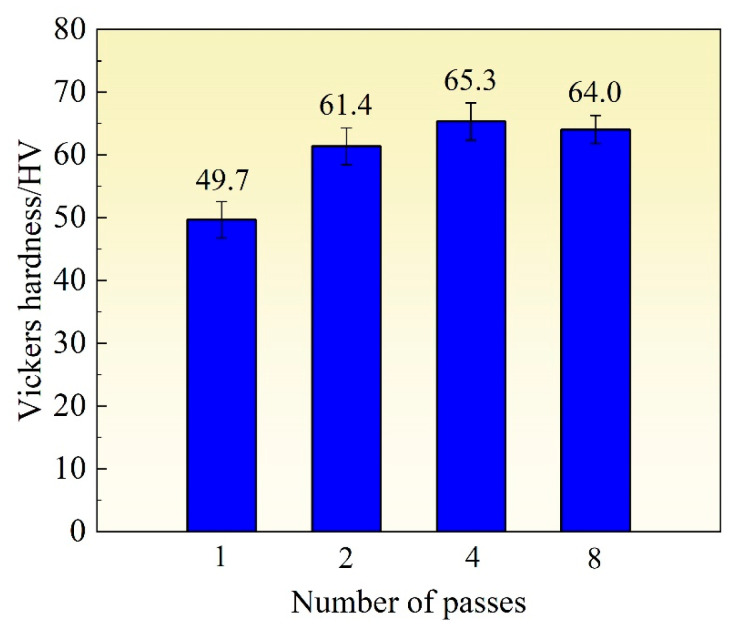
Microhardness of the alloy after different numbers of ECAP passes.

Figure 9 shows the microhardness of the alloy after different numbers of ECAP passes. To obtain representative values, microhardness was measured at ten randomly selected locations on each sample, and the average value was calculated. It is evident that the microhardness increases initially with the number of ECAP passes, reaching a maximum of 65.3 HV after four passes, and then decreases with further deformation. It is worth noting that although the microhardness of the alloy decreases after eight passes compared to four passes, the reduction is minimal. For micro-alloyed aluminum alloys, when the dislocation density is comparable, hardness is primarily determined by the grain size and the distribution of dispersed precipitate particles [29]. The average grain size of the alloy after four passes is significantly smaller than that after eight passes. However, the measured microhardness values differ only slightly. This discrepancy is likely due to the precipitation of relatively coarse precipitate particles along the grain boundaries after four passes, which weakens the overall precipitate strengthening effect [30,31].

#### 3.3.2. Tensile Strength

**Figure 10:**
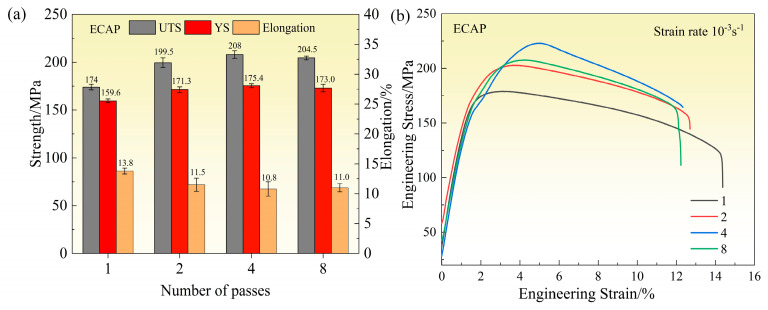
Tensile strength of the alloy under different passes: (**a**) tensile strength and (**b**) σ-ε curves.

Figure 10 shows the tensile properties of the alloy after different numbers of ECAP passes. As illustrated in Figure 10a, the ultimate tensile strength (UTS) initially increases with the number of ECAP passes, reaching a maximum value of 208 MPa after four passes. This represents an 82.3% improvement compared to the homogenization-annealed alloy (114.1 MPa). Beyond four passes, the UTS slightly decreases to 204.5 MPa at eight passes. The yield strength (YS) follows a similar trend, peaking at 175.4 MPa after four passes. The elongation remains above 10% under all ECAP conditions, indicating good ductility of the alloy.

The grain size reduction and shape change from elongated to more equiaxed grains contribute to the improved strength, with finer and more uniform grains hindering dislocation motion. However, after eight passes, the slight increase in grain size may lead to a small reduction in strength, suggesting an optimal grain size for achieving maximum tensile properties.

Figure 10b presents the engineering stress–strain curves, providing insight into the elastic and plastic deformation behavior of the alloy. All samples exhibit a well-defined linear elastic region with comparable slopes, suggesting that the ECAP process has a negligible effect on the elastic modulus. After necking, the one-pass sample shows the largest total elongation, likely due to a relatively low dislocation density and less developed grain boundary structure at this early deformation stage. In contrast, the samples subjected to two, four, and eight passes show a steeper rise to peak stress but a more rapid drop thereafter, suggesting enhanced strength but reduced post-necking ductility.

Overall, the ECAP process significantly improves strength while retaining adequate ductility. The observed differences in plastic deformation behavior reflect the complex interplay between grain refinement, dislocation evolution, and recrystallization behavior introduced by severe plastic deformation.

**Figure 11:**
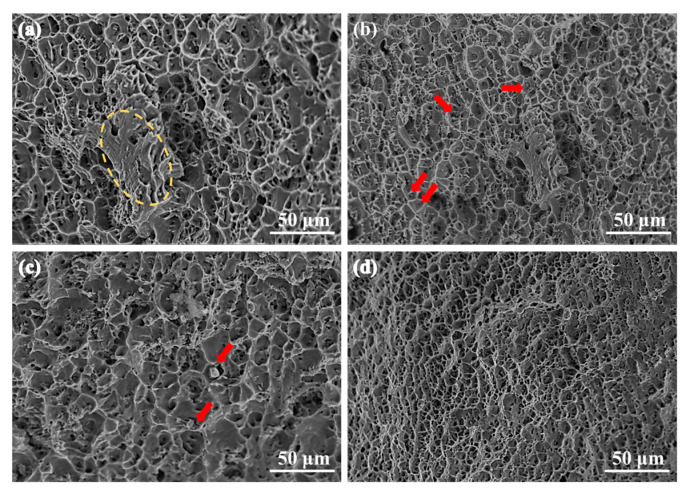
Fracture morphology: (**a**) 1p, (**b**) 2p, (**c**) 4p, (**d**) 8p.

Figure 11 shows the fracture morphology of the samples under different ECAP passes. It can be observed that the fracture surfaces of the ECAP-processed alloys are covered with a large number of dimples of varying sizes, indicating a ductile fracture mechanism. Notably, the fracture surfaces of samples processed with one and two ECAP passes also exhibit large, smooth regions (highlighted by yellow circles in the figure), showing distinct shear features. This may be attributed to the microstructural inhomogeneity introduced under these processing conditions [21]. In addition, numerous precipitate particles are observed at the bottom of the dimples, as indicated by the red arrows. In contrast, the dimples on the fracture surfaces of the alloys subjected to four and eight passes are more uniformly distributed.

#### 3.3.3. The Strengthening Mechanism of ECAP Alloy

The micro-alloyed aluminum alloy exhibits significant grain refinement after multi-pass ECAP processing, accompanied by a dispersed distribution of particles within the grains and a substantial increase in dislocation density. Compared with the as-cast alloy, its tensile properties are greatly improved. Therefore, it is necessary to analyze the strengthening mechanisms of the alloy subjected to different ECAP passes.

The yield strength ($\sigma_{YS}$) of the alloy comprises its inherent friction stress (a value of 10 MPa), solid-solution strengthening ($\sigma_{ss}$), grain boundary strengthening ($\sigma_{gbs}$), precipitation strengthening ($\sigma_{p}$), and dislocation strengthening ($\sigma_{dis}$), each with incremental contributions, as expressed in Equation (5) [32]:

(5) $$\sigma_{YS}=\sigma_{0}+\sigma_{ss}+\sigma_{gbs}+\sigma_{p}{+\sigma }_{dis}$$

Owing to the low solid solubility of Fe and Er in the Al matrix [33,34], the $\sigma_{ss}$ parameter can be ignored. As shown in Figure 4, the grain size of the alloy decreases, and the refined grains enhance the grain boundary strength, as calculated according to the Hall–Petch relationship expressed in Equation (6) [35]:

(6) $$\sigma_{gbs}=k_{1}d_{0}^{-\frac{1}{2}}$$

where $k_{1}$ is the Hall–Petch slope, and $d_{0}$ is the average grain size of the alloy. The $\sigma_{gbs}$ values of the multi-pass ECAP (1,2,4,8) alloy are approximately 30.9 MPa, 41.0 MPa, 72 MPa, and 59.9 MPa.

The dislocation density strengthening portion of the yield strength can be estimated from Equation (7) [36]:

(7) $$\sigma_{dis}=M\alpha Gb\rho^{1/2}$$

where M is the Talor factor of Al; α is the geometric constant (1.25 for Al); and ρ is the dislocation density, defined as

(8) $$\rho =16.1{\left(\varepsilon /b\right)}^{2}$$

where ε is the micro-strain of the sample obtained by XRD, which can be determined using Equation (9):

(9) βcosθ=kλ/d+4 εsinθ

where β is the true full width at half maximum (FWHM); λ is the wavelength of Cu Kα radiation (λ = 0.154 nm); k is a constant equal to approximately 0.9; θ is the Bragg angle; and d is the crystallite size. The physical meanings and values of the parameters used in the calculation are provided in Table 3.

According to the XRD patterns of the alloys in Figure 3, the values of ε obtained from the slope of the linear regression line are 0.00344%, 0.01302%, 0.0142%, and 0.00829%. Using this value to compute Equations (7) and (8) gives dislocation density of the alloy values of 0.2329 × 10^12^ m^−2^, 3.3366 × 10^12^ m^−2^, 3.9689 × 10^12^ m^−2^, and 1.3527 × 10^12^ m^−2^. The corresponding contributions of the dislocation density strengthening of the alloy are approximately 13.41 MPa, 50.76 MPa, 55.36 MPa, and 32.31 MPa.

**Figure 12:**
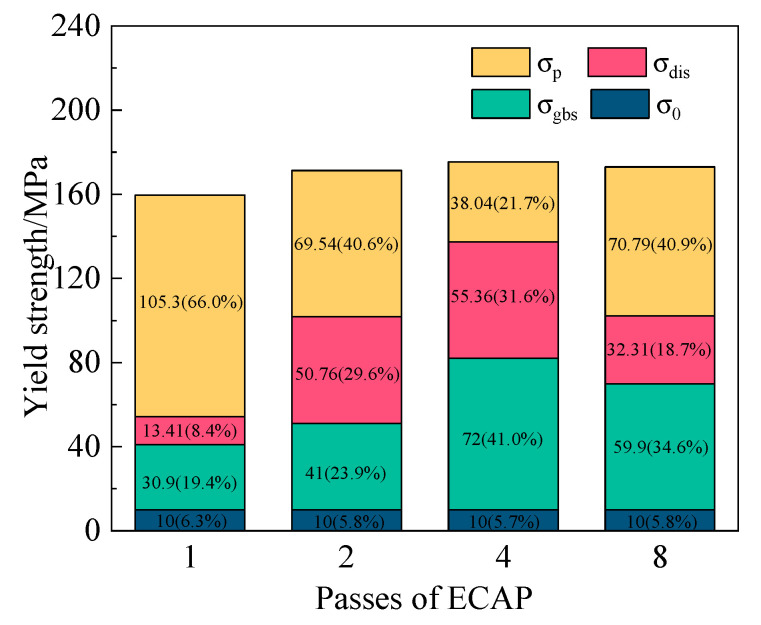
Contribution of strengthening mechanisms to yield strength of the micro-alloyed alloy during ECAP processing.

Accordingly, the contribution of precipitation strengthening ($\sigma_{p}$) in various alloys can be determined by the subtraction of the calculated results of grain boundary strengthening and dislocation density strengthening from the actual yield strength [37,38,39]. The phases, such as Al_3_Er, Al_13_Fe_4_, and Mg_2_Si, play a significant role in enhancing the yield strength through precipitation strengthening. These phases obstruct dislocation motion, acting as pinning points that prevent dislocations from easily moving through the matrix. The Al_3_Er phases, in particular, provide heterogeneous nucleation sites for grain refinement and also strengthen the material by promoting the formation of fine precipitates during the ECAP process [15]. The relative contributions of different strengthening mechanisms to the overall yield strength are illustrated in Figure 12.

**Table 3:** Physical meaning and values of parameters used in the calculation.

Parameter	Description	Value	Unit	Ref(s).
$k_{1}$	Hall–Petch coefficient	0.12	MPa × m^1/2^	[7]
G	Shear modulus	25.4	GPa	[40]
b	Burgers vector	0.286	nm	[40]
M	Talor factor	3.06	Dimensionless	[41]
λ	Wavelength of Cu Kα radiation	0.154	nm	[42]

The nonlinear superposition of individual contributions due to coupling effects of multiple strengthening mechanisms may be the reason for the difference between the measured and calculated results. Nevertheless, the results indicate that precipitation strengthening is the primary mechanism contributing to the yield strength enhancement of the ECAP alloy.

### 3.4. Effect of ECAP on Conductive Properties

**Figure 13:**
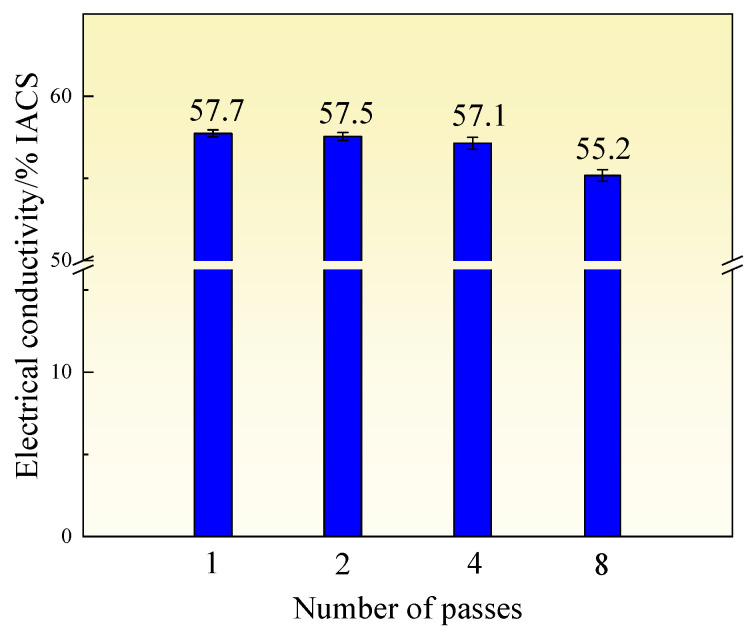
Electrical conductivity of the alloy after different ECAP passes.

Figure 13 shows the electrical conductivity of the Al-0.7Fe-0.4Mg-0.1Si-0.5Er alloy after different numbers of ECAP passes. The electrical conductivity of the alloy exhibits a decreasing trend with increasing ECAP passes. When the number of passes is less than or equal to 4, the conductivity remains relatively high, exceeding 57%IACS. However, when the number of passes increases to 8, the conductivity decreases significantly, dropping to 55.2%IACS. This reduction is primarily attributed to the increased density of dislocations, grain boundaries, and precipitates within the alloy after ECAP passes which create scattering sites for electrons, thus contributing to higher electrical resistivity [28,43,44].

## 4. Conclusions

In this study, the effects of multi-pass equal channel angular pressing (ECAP) on the microstructure and properties of an Er-micro-alloyed Al-0.7Fe-0.4Mg-0.1Si alloy were systematically investigated. The main conclusions are as follows:

(1) ECAP effectively refined the grain structure and promoted the precipitation of phases. Grain refinement was primarily attributed to CDRX and GDRX induced by severe plastic deformation.
(2) With increasing ECAP passes, the average grain size first decreased and then slightly increased, while the precipitate fraction exhibited the opposite trend, initially increasing due to deformation-induced precipitation and then declining due to partial dissolution and coarsening at higher passes.
(3) The alloy exhibited excellent mechanical properties and electrical conductivity after ECAP, resulting from the combined effects of grain refinement strengthening, dislocation strengthening, and precipitate strengthening. The optimal performance was achieved after four passes, with a tensile strength of 208 MPa and an electrical conductivity of 57.1%IACS.
(4) This work provides a comprehensive understanding of the microstructure–property relationship in Er-micro-alloyed aluminum alloys under multi-pass ECAP, offering practical insights into optimizing the strength–conductivity balance in micro-alloyed systems.

Further studies will compare conventional heat treatments with combined post-ECAP heat treatments for alloys with different Er contents to enhance performance while maintaining the strength–conductivity synergy.

## Figures and Tables

**Table 1:** Chemical composition of Al-0.7Fe-0.4Mg-0.1Si-0.5Er (wt.%).

Elements	Fe	Si	Mg	Cu	Zn	B	Er	Al
content	0.75	0.1	0.37	0.04	0.05	0.03	0.47	Bal.

**Table 2:** Precipitate statistics after different ECAP passes.

Precipitation	1p	2p	4p	8p
Al	97.2%	95.9%	84.5%	94.4%
β″ (Mg_5_Si_6_)	0.7%	1.8%	6.6%	2.4%
β′ (Mg_6_Si_3.3_)	0%	0%	0.1%	0%
β (Mg_2_Si)	0.7%	0.8%	3.4%	1%
Al_13_Fe_4_	1.5%	1.5%	5.4%	2.2%

## Data Availability

The original contributions presented in this study are included in the article. Further inquiries can be directed to the corresponding author.
